# Hydrodynamic performance of suction feeding is virtually unaffected by variation in the shape of the posterior region of the pharynx in fish

**DOI:** 10.1098/rsos.181249

**Published:** 2018-09-19

**Authors:** Pauline Provini, Sam Van Wassenbergh

**Affiliations:** 1 Département Adaptations du Vivant, UMR Mécanismes adaptatifs et évolution (MECADEV), Muséum National d'Histoire Naturelle/CNRS, 57 rue Cuvier, Case Postale 55, 75231 Paris Cedex 05, France; 2 Department of Biology, University of Antwerp, Universiteitsplein 1, 2610 Antwerp, Belgium

**Keywords:** intra-oral streamlining, prey capture, biomechanics, computational fluid dynamics

## Abstract

To capture prey by suction, fish generate a flow of water that enters the mouth and exits at the back of the head. It was previously hypothesized that prey-capture performance is improved by a streamlined shape of the posterior region of the pharynx, which enables an unobstructed outflow with minimal hydrodynamic resistance. However, this hypothesis remained untested for several decades. Using computational fluid dynamics simulations, we now managed to quantify the effects of different shapes of the posterior pharynx on the dynamics of suction feeding, based on a feeding act of a sunfish (*Lepomis gibbosus*). In contrast to what was hypothesized, the effects of the imposed variation in shape were negligible: flow velocity patterns remained essentially identical, and the effects on feeding dynamics were negligibly small. This remarkable hydrodynamic insensitivity implies that, in the course of evolution, the observed wedge-like protrusions of the pectoral surfaces of the pharynx probably resulted from spatial constraints and/or mechanical demands on the musculoskeletal linkages, rather than constraints imposed by hydrodynamics. Our study, therefore, exceptionally shows that a streamlined biological shape subjected to fluid flows is not always the result of selection for hydrodynamic improvement.

## 1. Introduction

The physical interactions between organisms and their surrounding fluid environments are critical in the evolution of form, function, behaviour and physiology. These interactions lay the foundation for behaviours as diverse as flying, swimming, breathing and feeding [1]. Performance in each case has a decisive impact on the survival and reproduction of individuals, and consequently on the evolution of species. Several studies have shown how the evolution of adapted morphology and body kinematics that match the dynamic properties of fluids have enabled certain species to have highly efficient swimming or flying performances (e.g. [2–4]). Still, for many vital elements of animals, it remains unknown how the shape is tuned to fluid-dynamic performance.

One of these elements is the buccopharyngeal cavity of fishes. To capture food, most fishes and other vertebrates that feed underwater, rely on an abrupt expansion of their buccopharynx (i.e. the water-filled, internal volume of the head which spans from the mouth to the oesophagus entrance). This expansion causes a flow of water relative to the head. This water enters the mouth to fill the expanding intra-oral volume and to carry food towards the posterior end of the buccopharynx (e.g. [5]), where food can subsequently be masticated (e.g. by pharyngeal jaw action [6]) and forced into the oesophagus. The opening of the external gill slits, which form the opercular and branchiostegal valves, allows water to exit the buccopharyngeal cavity. By allowing continuation of the flow of the previously sucked water in the posterior direction relative to the head, this *outflow* also enables a faster and prolonged *inflow* into the mouth when the mouth is closing [7]. The importance of the outflow phase for predatory suction feeders has been clearly demonstrated in salmon (*Salmo gairdneri*), for which it was shown by three-dimesional flow visualization that the volume of water that is sucked into the mouth *after* the opening of the opercular and branchiostegal valves is 5.5 times larger than the sucked volume *before* this valve opening [8]. However, while the biomechanics of suction feeding has been intensively studied (e.g. reviewed in [9,10]), the influence of variation in the shape of the intra-oral cavity on suction-feeding hydrodynamics has not been a focus of research.

**Figure 1.**
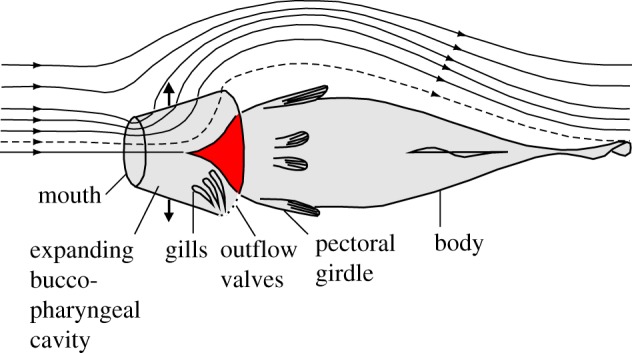
Illustration of Muller & Osse's hypothesis on intra-oral streamlining (modified after Muller & Osse [5]). For a suction-feeding fish in ventral view with the head modelled as an expanding (see arrows) truncated cone, both calculated streamlines passing into the mouth and external of the fish are shown, with the dashed streamline continuing through the opercular and branchiostegal valves. Note that because the head expands, also other streamlines will ‘cross’ the buccopharyngeal cavity wall. The conical wedge-like shape of the pectoral girdle, hypothesized to be important for minimizing hydrodynamic resistance, is highlighted in red.

Nevertheless, several decades ago, Muller & Osse [5] proposed a hypothesis on intra-oral streamlining in fish. This hypothesis states that a conical wedge inside the pharyngeal cavity, formed by the pectoral girdle, helps to minimize hydrodynamic resistance in fish species where an unhampered flow is important (figure 1). The shape of the posterior wall of the pharynx would minimize the local changes in flow direction during suction, thereby maximizing the preservation of momentum of the flow in the posterior direction. The benefits of the hypothetically reduced flow resistance due to intra-oral streamlining would be twofold: (i) faster anterior-to-posterior suction flows can be produced (implying also a larger volumetric inflow into the mouth during the phase with opened gill slits) and (ii) the approaching predator would lose less of its forward speed needed to overtake prey due to drag forces. In this respect, the posterior border of the pharynx can be regarded as the front end of a body that faces a water flow during suction feeding. This body then consists of the pectoral girdle, the trunk and tail of the fish (figure 1), and fluid-dynamic principles would be similar to those of an external flow over this body, for which drag reduction due to streamlining will apply.

The objective of this article is to test Muller & Osse's hypothesis on intra-oral streamlining [5]. How important is it for fish to have a wedge-like protrusion in the pharynx to improve suction-feeding performance? What aspects of the dynamics of suction feeding are affected by the shape of the posterior wall of the pharynx? Answering these questions will be crucial for our understanding of the (evolutionary) functional morphology of the head of fishes, as it will unravel whether hydrodynamics place constraints on the shape of the buccopharyngeal cavity, and how strong these constraints are.

## 2. Material and methods

### 2.1. Modelling approaches

To quantify the effects of morphological variation in the posterior region of the pharynx during suction feeding, simulations of suction feeding were performed using the method of computational fluid dynamics (CFD). This technique numerically solves the equations of motion of fluids (i.e. Navier–Stokes equations for momentum conservation) while maintaining continuity of flow (i.e. mass conservation) in a certain domain for which different types of boundary conditions can be specified. This technique is typically used to study the influence of variation in shape on performance in a fluid-dynamic context when analytic solutions do not exist, or physical modelling is not practically possible (e.g. [11]). This situation clearly applies to the fast, cranial movements that fish use to generate suction.

**Figure 2.**
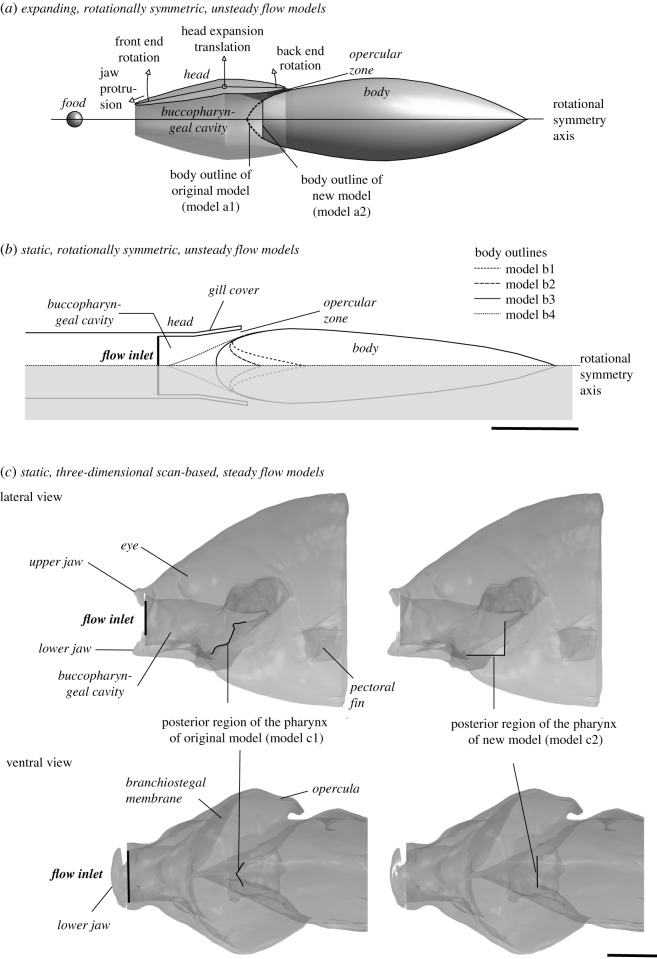
The three types of CFD models used in this study. (*a*) *Expanding, rotationally symmetric, unsteady flow models*, based on an existing model [12] in which the head is subjected to expansion and compression by four equations (translation, front-end rotation, back-end rotation, jaw protrusion), the profile of the posterior region of the buccopharyngeal cavity is modified by truncating its rounded shape (i.e. the front end of the ‘body’). (*b*) *Static, rotationally symmetric, unsteady flow models*, describing four different shapes of the pectoral region of the pharynx with open gill slits, while a short flux of water is forced to enter the mouth. (*c*) *Static,* *three-dimensional* *scan based, steady flow models,* based on an X-ray computed tomography scan of a *Lepomis gibbosus* specimen, with water forced to enter the mouth. The posterior wall of the pharynx is either unmodified or flattened. Scale bars, 10 mm.

Three complementary approaches will be used (figure 2). A first approach is to use an existing model of suction feeding in a sunfish (*Lepomis gibbosus*) [12] (figure 2*a*). To date, this model is the most complete in its inclusion of the different dynamic aspects of suction feeding: the expansion of the buccopharyngeal cavity is modelled as an anterior-to-posterior wave of expansion using three equations, protrusion of the jaws is included, unsteady forward movement towards the food is accounted for, and the opening of the gill slits is modelled to allow outflow of water [12]. The original shape of this model, in which the pectoral region forms the front of a bullet-shaped body that resembles Muller & Osse's hypothetical optimal shape [5], will be modified into a shape that is presumably less streamlined (figure 2*a*). The disadvantages of this model are its high computational costs, and its simplified, rotationally symmetric shape.

A second approach overcomes the computational time of the model described above and will be used to further explore the effects of differences in the shape of the pectoral region of the pharynx (figure 2*b*). The range of shapes will be larger, and to evaluate whether the shape described by Muller & Osse [5] is optimal, will go beyond the biologically observed forms. Here, the focus will be on the phase of outflow of water from the buccopharyngeal cavity, and the associated intra-oral flows at this instant. The model will be fixed in a position with open gill slits, while a short flux of water will be forced to enter the mouth. This model will also allow us to evaluate the generality of the result from the more complex, expanding model (figure 2*a*).

A third approach overcomes the geometrical simplifications of the previous models (figure 2*c*). This three-dimensional model will be based on an X-ray computed tomography scan of a *Lepomis gibbosus* specimen. The scanned specimen will be fixed in a position with opened opercula and branchiostegal membranes, mimicking the instant of strongest outflow during powerful suction. Like the second approach, water will be forced to enter the mouth, will flow through the mouth cavity and will exit through the slits between, on one side, the opercula and branchiostegal membranes, and on the other side the pectoral region of the lateral side of the body. Modifying the shape of the posterior wall of the pharynx in a similar way to that in the first approach (figure 2*a*) will reveal how three-dimensional flows are influenced, and whether this differs from the rotationally symmetric models. However, as this three-dimensional model remains static (i.e. no expanding buccal volume is modelled) and subjected to a water flow that is constant in time, the models from the first two approaches are required to evaluate the effects of potential changes in flow patterns in this three-dimensional model on the dynamics of suction feeding.

Note that in all these models, we assume the effect of the branchial arches and gills (i.e. the gill basket) to be negligible, and these structures are excluded from the models. Although these elements will inevitably cause an (unknown) amount of hydrodynamic resistance, there are several reasons to assume that their influence on the patterns of suction flows is not large. First, a wide expansion of the buccopharyngeal cavity during suction feeding will extend the initially folded branchial arches, so that space in between the consecutive arches increases [13]. Large spaces in between the gills are shown in still images from high-speed videos at the instant of maximal expansion of the head in a diversity of suction-feeding fishes (e.g. [5]). Second, the typically hydrofoil-shaped branchial arches together with the flexible gill filaments that will align with high-velocity flows seem morphologically well-adapted to minimize hindrance to the powerful flows during suction feeding [5]. In the case of subtle effects on flow dynamics, these effects will be similar in all models and therefore not interfere with our analysis. Consequently, we consider it justified to simplify our models by the exclusion of the branchial basket.

For each type of model, the geometry was designed in ANSYS DesignModeler 18.0 (Ansys Inc., Lebanon, NH, USA), meshing was performed in ANSYS Meshing 18.0, and laminar flows of water (at 20°C: density 998.2 kg m^−3^; viscosity 1.003 mPa s) were calculated in ANSYS Fluent 18.0. Laminar flow models were chosen because the transition to turbulent flows is unlikely to be reached in relatively small suction feeders [14]. In Fluent, we used the pressure-based solver (chosen to obtain fast-converging solutions), the standard pressure discretization scheme for the pressure calculation and a second-order upwind scheme for momentum equations. The pressure–velocity coupling was solved using the robust, default SIMPLE scheme. The latter is a discretization method that uses a relationship between velocity and pressure corrections to enforce mass conservation and to obtain the pressure field.

### 2.2. Expanding, rotationally symmetric, unsteady flow models

This model is described in detail in a previous publication [12]. In short, this is an axially symmetric CFD model of a suction-feeding sequence by the sunfish *Lepomis gibbosus* (total length = 76 mm) capturing a freely suspended bloodworm. The measured kinematics of head expansion and jaw protrusion is approximated by four equations (figure 2*a*). The food was modelled as a sphere of 1 mm radius of which the distance to the centre of the mouth will match that of the bloodworm at the onset of head expansion (figure 1). The model's forward speed to approach the prey is initially prescribed to reach a velocity of 0.1 m s^−1^, observed swimming velocity, during the first 30 ms of the simulation, was moved at constant velocity for another 5 ms after which the axial acceleration of the fish was calculated with Newton's second law of motion. To do so, axial forces on the head and body were calculated and summed after each time step, and by accounting for the mass of the fish, used to calculate the fish's velocity in the next time step. The movement of the food (assumed to have the same density as the water) is calculated in the same way. To avoid an inflow of water through the gill slit while suction is generated (i.e. when pressure in the buccopharyngeal cavity is negative), viscosity of a separately defined fluid entity near the opercula (the ‘opercular zone’ in figure 2*a*) is controlled to block early inflow (high viscosity assigned) and later outflow can be allowed (changing viscosity to that of water when local pressure becomes positive). Apart from using the kinematics of the original model, also a model with a considerably increased approaching speed (0.5 m s^−1^) and expansion amplitude (+50%) is subjected to the pharyngeal modification treatment. The latter model will be representative of a ram-feeding fish.

### 2.3. Static, rotationally symmetric, unsteady flow models

The geometry of this model is based upon the dimensions of *Lepomis gibbosus* with open mouth and expanded opercular valves (figure 2*c*). From a ventral view of the fish, we drew the outlines of the mouth, the opercular valves and pectoral region as a series of spline curves. The position of the control points of the splines was defined by parameters to adjust the shape of the model, such as the axial position of the gill cover, the angle of gill cover, the width of the buccopharyngeal cavity, the length of the gill cover, the axial position of the oesophagus entrance point. The diameter of the body and the distance between the mouth and the body were kept constant.

Geometries of four different shapes were imported in Ansys Meshing and meshed with approximately 0.5 million triangular cells. The surface area of the mouth aperture was defined as a flow inlet with a prescribed velocity. At this inlet, we enforced a flow velocity rising up from 0 to 1 m s^−1^ in 20 ms, after which a constant velocity of 1 m s^−1^ was maintained. Flows were solved for 0.2 s with time steps of 0.5 ms. Two hundred iterations per time step were sufficient to reach a converged solution. Mesh convergence was tested by monitoring the drag force on the model for meshes of different sizes. While a considerable difference was noted for the change from a mesh with 0.14 million to 0.25 million cells (−11%), this change decreased to +2.5% with a consecutive refinement to the selected mesh of 0.5 million cells.

### 2.4. Static, three-dimensional scan based, steady flow models

One individual of *Lepomis gibbosus* was bought from a commercial dealer and fixed in a 4% paraformaldehyde and 2% glutaraldehyde solution, with an open mouth and expanded opercular and branchiostegal valves. In order to image the animal soft tissues, the specimen was subsequently transferred in a contrast stain solution of 2.5% phosphomolybdic acid for 10 days [15] and scanned at the AST-RX Platform (Accès Scientifique à la Tomographie à Rayons X) of the Muséum National d'Histoire Naturelle, Paris. We obtained the tomography projection images needed to reconstruct the cross-section images with NRecon volumetric reconstruction software (SkyScan, 1.4.0, Kontich, Belgium). Then, the Avizo software (v. 6.3; FEI Visualization Sciences Group) allowed us to reconstruct the three-dimensional model of the fish, especially the external three-dimensional shape of the head and pectoral region, as well as the buccal cavity. The three-dimensional model was imported in Geomagic DesignX (3D Systems, Morrisville, NC, USA) for cleaning and generating spline surface patches that can be imported in DesignModeler. In DesignModeler, we defined the geometry of the fluid domain by creating a sphere of 0.5 m radius centred on the initial geometry of the fish model. Then, a cylinder of 0.006 m radius was connected to the mouth aperture of the fish. We applied a Boolean subtraction to obtain a single body containing the cylinder and the fish head inside the sphere. On a second model, named the flat model, we added a box of 0.0078 m × 0.012 m × 0.008 m inside the mouth, at the level of the back of the oral cavity. We performed a Boolean subtraction to obtain one body with a flat back of the mouth. Geometries were imported in Meshing software and meshed with 11.6 million tetrahedral cells. Further refinement to a mesh of 15.2 million cells only had a negligible influence on drag force (+0.6%). At the level of the mouth, we imposed a flow inlet with a prescribed velocity of 0.5 m s^−1^.

## 3. Results

### 3.1. Expanding, rotationally symmetric, unsteady flow models

**Figure 3.**
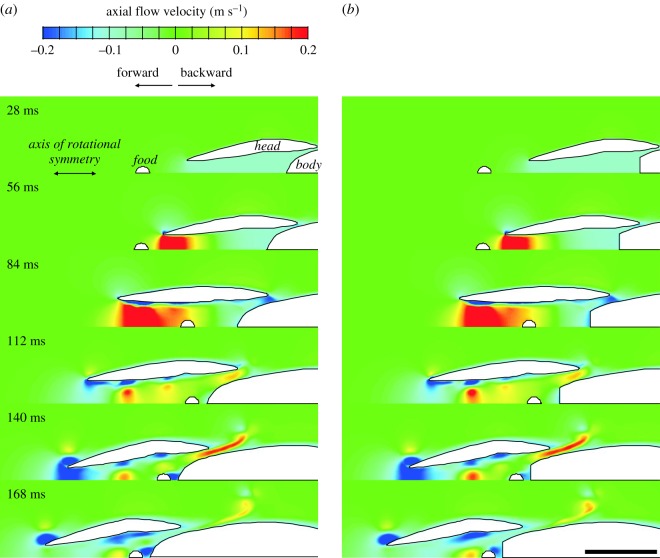
Effect of truncation of the pectoral region of the pharynx on axial flow velocity patterns in a dynamically realistic model of suction feeding. (*a*) The original model of a food-capturing sunfish (*Lepomis gibbosus*) from Van Wassenbergh [12] is shown alongside a second model (*b*) with the same expansion kinematics of the buccopharynx but with a truncated pharynx. Note that the overall patterns of flow are similar. Scale bar, 10 mm.

Similar overall flow patterns were observed in the expanding, rotationally symmetric, unsteady flow models (figure 3). During the phase of suction (between simulation times of 40 ms and 70 ms) as well as afterwards when the water exited the opercular slits (maximal velocity around simulation time of 130 ms), axial flow velocity plots showed an almost identical image for the original model (figure 3*a*) and the model with the truncated posterior pharynx (figure 3*b*). Both models also showed a similar ring vortex due to the existing water travelling posteriorly during the final instants of the simulation.

**Figure 4.**
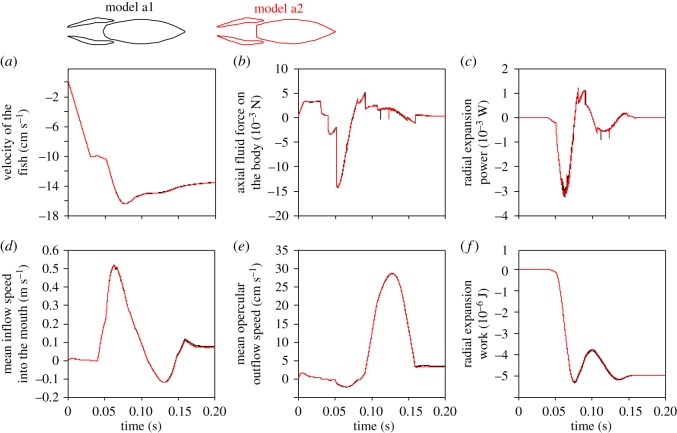
Hydrodynamic differences between two models with a different shape of the pectoral region of the pharynx during a strike with a slow approach of 0.1 m s^−1^. Note that the data curves of the two models (model a1 = black; model a2 with truncated pharynx = red) generally overlap. This is shown for (*a*) the axial velocity of the fish (which is calculated based the hydrodynamic forces on the fish starting from simulation time 0.035 s), (*b*) the forces exerted by the water on the body, (*c*) the power required to expand the head, (*d*) the mean speed of water inflow into the mouth, (*e*) the outflow rate through the model's gill slit and (*f*) the work (cumulating from time 0) to overcome hydrodynamic resistance for radial expansion. In (*c*) and (*f*), negative values denote power and work required to be generated by the fish. Positive (axial) velocities or forces in (*a*, *b*, *d* and *e*) are directed posteriorly. Inflow (*d*) and outflow (*e*) velocities are calculated with respect to the fish-bound frame of reference.

The forward velocity of the fish, which was calculated based on the hydrodynamic forces in the axial direction exerted on the fish from simulation time 35 ms onward, increased up to a peak speed that was 0.13% higher in the truncated model, while the final speed (i.e. at simulation time 0.2 s) was 0.58% higher in this model (figure 4*a*). The force exerted by the water on the modified body (model a2) in the axial direction was 1.5% higher in magnitude at the instant it reaches its (negative) peak compared to the original model (model a1). At the instant of maximal outflow speed, forces were 6.8% higher (figure 4*b*). The maximal power required to expand the head was virtually identical (−0.018% difference; figure 4*c*), resulting in slightly smaller mechanical work required from the muscles (−0.39% difference; figure 4*f*). The mean inflow rate into the mouth with respect to the moving fish was 0.35% higher at instant of peak inflow speed, and 6.5% smaller at the end of the simulation (figure 4*d*). The mean outflow rate from the opercular slits was 0.19% smaller at peak outflow speed, and 6.5% smaller at the end of the simulation. Based on the transition of pressure from negative to positive at the opercular region of the buccopharyngeal cavity, opercular valve opening was predicted to occur 0.2 ms earlier (i.e. a 0.36% difference in expansion time with closed opercular valves).

**Figure 5.**
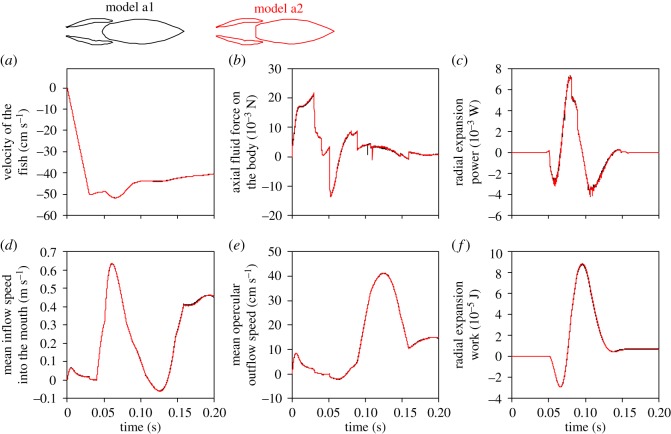
Hydrodynamic differences between two models with a different pectoral region of the pharynx during a strike with a fast approach of 0.5 m s^−1^ and 50% increased expansion amplitude. Similar to the strike with a considerably slower approaching speed (figure 4), the data curves of both models (model a1 = black; model a2 with truncated pharynx = red) remain in close proximity. See the legend of figure 4 for notes on the meaning of the reported variables.

Under the conditions of ram-feeding kinematics, the differences between the models did not change notably (figure 5). Comparing the modified model (model a2) relative to the original model (model a1), the peak forward velocity was 0.061% higher and the final velocity 0.58% higher (figure 5*a*). The force exerted by the water on the body was 2.5% higher in magnitude at the negative peak, and 12% at the final instants of the simulation (figure 5*b*). The maximal power required to expand the head was 0.41% higher (figure 5*c*). Although the entire act required no network investment for radial expansion, from the start to the instant of peak suction power (simulation time = 60 ms), model a2 required 0.55% less energy input to perform its radial expansion. The mean inflow rate into the mouth was 0.57% smaller at the instant of peak inflow speed, and 0.40% higher at the end of the simulation (figure 5*d*). The mean outflow rate from the opercular slits was 0.28% smaller at peak outflow speed, and 0.86% higher at the end of the simulation (figure 5*e*). Opercular valve opening was predicted to occur 0.3 ms later (i.e. a 0.56% difference in expansion time with closed opercular valves).

### 3.2. Static, rotationally symmetric, unsteady flow models

**Figure 6.**
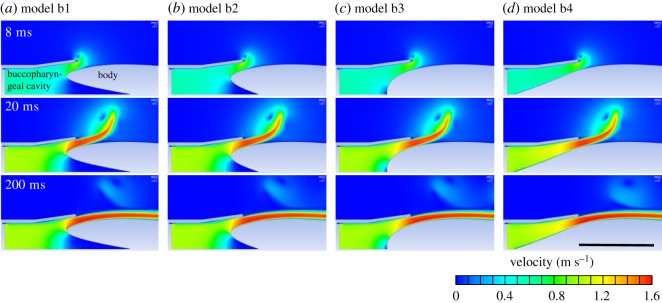
Effect of the shape of the pectoral region of the pharynx on flow velocity patterns in four static, rotationally symmetric, unsteady flow models. (*a–d*) At three different time steps of the simulation (*t*_1_ = 8 ms, *t*_2_ = 20 ms, *t*_3_ = 200 ms). Note that the overall patterns of flow are similar. Scale bar, 10 mm.

Small differences between flow patterns were observed among the four static rotationally symmetric models with equal inlet velocity prescribed at the mouth (figure 6). Flow velocities were remarkably similar over the entire domain in the different body outlines observed at three simulation times, i.e. at 8 ms (t1), 20 ms (t2) and 200 ms (t3) (figure 6; electronic supplementary material, table S1). The maximum value of velocity was extracted for each model at these three instants and compared to model b3. Since the latter model is considered as a reference for its similarity to the original, expanding, rotationally symmetric model a1, all subsequent differences are expressed relative to b3. We obtained flow velocity differences inferior to 1.5% for each model at t1, t2 and t3. The largest difference was observed for b1 at simulation time 8 ms (0.99 m s^−1^ compared to 0.98 m s^−1^ in b3), but this difference decreased to 0.02% at simulation time of 20 ms.

**Figure 7.**
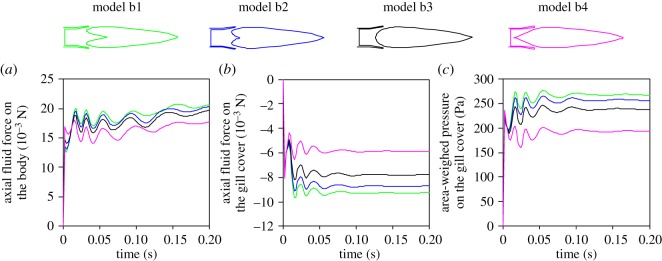
Effect of the shape of the pectoral region of the pharynx in four static, rotationally symmetric, unsteady flow models on the axial fluid force on the body (*a*), on the gill cover (*b*) and on the net pressure on the gill cover (*c*).

The axial fluid force exerted on the body was computed for each model through time (figure 7*a*). Model b2 showed a maximum difference of 7.2% from our reference model b3 at the beginning of the simulation (at t1 the force on the body was 0.013 N for b2 compared to 0.014 N for b3), which rapidly decreased to around 4% of difference at t2, after 8 ms of simulation. Models b1 and b4 presented a larger relative difference, with a decrease in axial force on the body of 5% in b1 compared at t2 (0.0137 N and 0.0144 N, respectively) and a decrease in axial force on the body of 9% in b4 at t2 (0.0157 N versus 0.0144 N in b3). The differences between models at specific instants reported above are larger than the overall effect of the force on the body acting over the entire simulation: the impulse calculated for each model ranged from −7% to +6% compared to b3 (impulse of 3.75 mN s, 3.65 mN s, 3.53 mN s and 3.26 mN s for b1, b2, b3 and b4, respectively). The axial fluid force exerted on the gill cover was higher in model b4 compared to the other three models (figure 7*b*). The impulse on the gill cover, calculated for each model for the entire simulation, ranged from −24% to +18% compared to b3.

The area-weighted average of pressure on the gill cover was lower in model b4 compared to the other models (figure 7*c*; mean pressure of 260.5 Pa, 250.3 Pa, 232.3 Pa, and 190.3 Pa for b1, b2, b3 and b4, respectively). Differences in pressure at the anterior part of the buccopharyngeal cavity were small, with a difference of the maximum value of pressure on the head surface inferior to 4.0% at simulation times 8 ms (t1), 20 ms (t2) and 200 ms (t3) for each model compared to model 3 (figure 2*b*; electronic supplementary material, table S1). The main differences appeared at the axial part of the pharynx, anterior to the posterior pharynx wall, revealing a larger zone with low flow velocity, especially when the shape was not streamlined (models b1 and b2; figure 6).

### 3.3. Static, three-dimensional scan based, steady flow models

**Figure 8.**
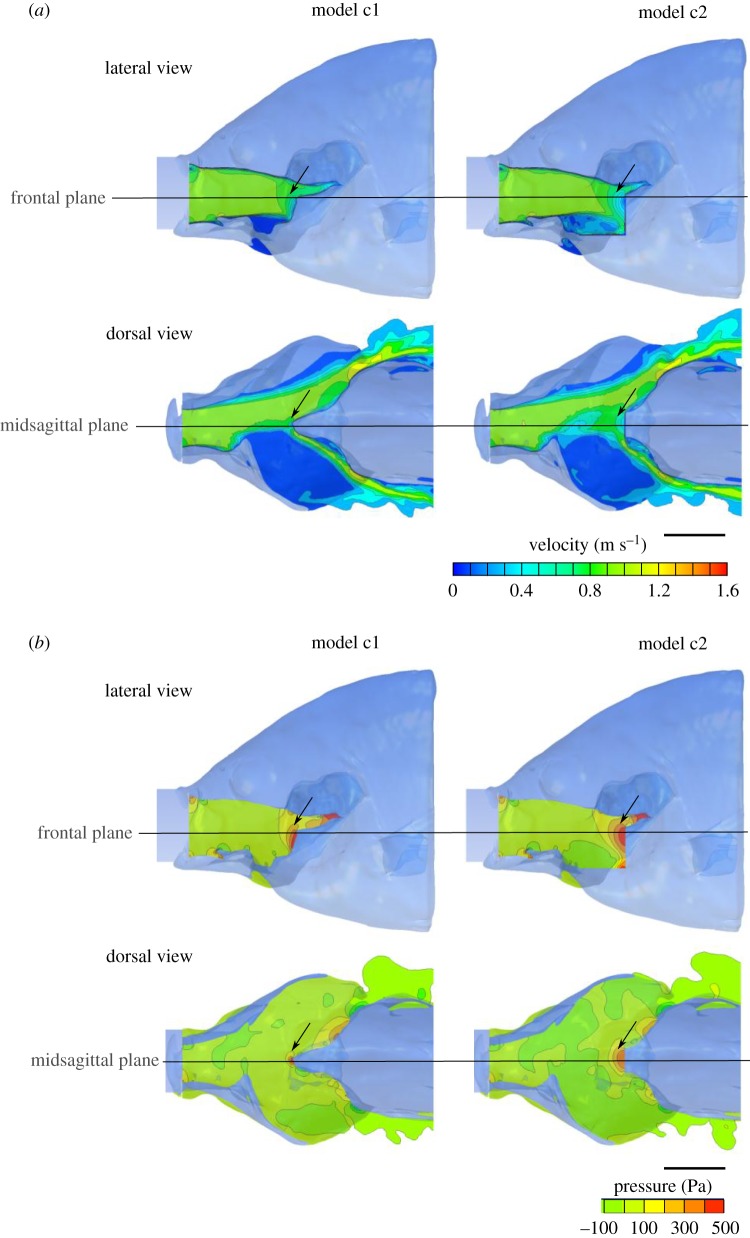
Effect of truncation of the pectoral region of the pharynx on the static, three-dimensional scan based, steady flow models. (*a*) The three-dimensional velocity magnitude and (*b*) pressure on the midsagittal and a frontal plane of the original model of a sunfish (*Lepomis gibbosus*), with mouth and gill slits open, is shown alongside a second model with the same three-dimensional position but with a truncated pharynx. Note that the overall patterns of flow are similar, except directly upstream from the truncated region (arrows on the figure). Scale bars, 10 mm.

The central area of the buccal cavity showed similar velocity contours and magnitudes between the original and the truncated model (figure 8*a*). The maximum value of velocity calculated on the original model c1 was 1.28 m s^−1^ compared with 1.32 m s^−1^ in model c2 on the midsagittal plane (3.0% difference), and 1.21 m s^−1^ compared with 1.22 m s^−1^ on the frontal plane (1.5% difference).

Similarly, the overall pressure patterns were comparable between the two models, with a relatively high-pressure zone at the posterior part of the pharynx, where the pressure reached magnitudes superior to 200 Pa (figure 8*b*; electronic supplementary material, table S2). Even if this zone was larger in c2 compared with c1, we measured similar maximum values of pressure on the midsagittal plane of both models, with 494.6 Pa and 496.5 Pa, respectively (0.38% difference; figure 8*b*; electronic supplementary material, table S2).

## 4. Discussion

Our results did not support the hypothesis proposed by Muller & Osse [5], who stated that streamlining of the posterior region of the pharynx, ideally into wedge-like or bullet-like shapes, is important to optimize the flow of water through and out of the buccopharyngeal cavity during suction feeding in fishes [5]. By contrast, our CFD models showed a remarkable similarity in flow patterns between models with a variety of shapes in this region (figures 3, 6 and 8), for feeding acts with both slow and fast approaches. A streamlined shape of the posterior region of the pharynx is thus not a requirement for fast and unidirectional flows during suction feeding. Also, the dynamics and energetics of the movements performed by the suction feeder remained virtually unaffected by these changes in shape. Drag forces on the body of the fish remained virtually identical (figures 4*b* and 5*b*), and so did the associated deceleration of the fish during the outflow phase of suction (figures 4*a* and 5*a*). The expansion and compression of the buccopharyngeal cavity did not require more power (figures 4*c* and 5*c*) or work (figures 4*f* and 5*f*) when a bullet-like posterior pharynx is modified into a flat surface. The models predicted no change in the timing of opercular valve opening. Consequently, suction-feeding hydrodynamics appears to be virtually insensitive to shape changes in the pectoral region of the pharynx.

Difference between models could be considered as biologically relevant when comparing extreme variations in pharynx shape. This was the case for the model with the extremely long and sharply protruding posterior wall of the pharynx (model b4; figure 2*b*). The largest difference was noted in the forces on the gill cover, which were smaller in model b4 (figure 6*b,c*). This force will cause abduction of the gill cover, and (in a dynamic setting) could influence the way water is jetted out of the opercular and branchiostegal slits. However, it would probably require a slight stiffening of the gill cover and/or, for example, an increased stretch resistance or earlier activation onset of the gill cover's adductor muscles to cancel out this effect on gill cover movement, and thereby preserve virtually identical flow patterns (as displayed in figure 6). Given the drastic variation in pharynx shape, the differences in drag force on the body were still relatively small (less than 10%; figure 7*a*). Considering the exaggerated variation in this set of models (figure 2*b*) to illustrate potential differences more clearly, the noted differences were still small enough to conclude that the selective pressures on shape changes to the pharynx (to optimize suction flows) are low.

**Figure 9.**
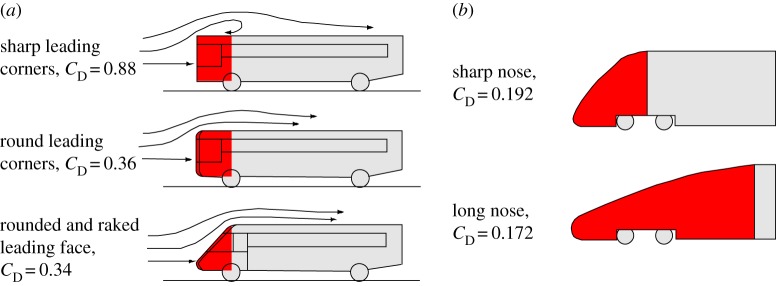
Effects of front-end shape modifications on drag forces in road and railroad vehicles. (*a*) The front-end shapes of buses (in red) can be significantly ‘streamlined’ by sufficiently rounding the corners (drag reduction by almost 60% from top to middle shape), while further changing the frontal angle into a more wedged shape has a minimal effect on streamline pattern and drag force (drag reduction by 5% from middle to bottom shape) (adapted from [17]). (*b*) A drag reduction of the same order of magnitude (about 10%) was measured for extreme nose elongation in high-speed trains (adapted from [20]). The results are consistent with the findings reported in figure 6.

The current results may be equivalent to the relatively small differences in fluid dynamics that are generally found for shape modifications at the front (i.e. the flow-facing side) of rigid bodies exposed to external fluid flows. The effects on drag reduction, for example, are typically much larger at the rear end, where flow separation occurs [16]. This has been extensively studied in the context of the aerodynamic design of road and railroad vehicles. Unless there are sharp edges, the degree of ‘rounding’ of the front end of domestic cars results in very little if any drag reduction [17]. While intuitively, it may be imagined that inclining the angle of an initially vertical front window (i.e. raking the front face) would make the vehicle more streamlined, substantial changes in the total drag force are typically absent [18]. Studies on the aerodynamics of buses clearly illustrate that the drag penalty of front-end changes other than illuminating sharp edges is minimal [19] (figure 9*a*). Likewise, relatively low drag reduction is measured for extreme nose elongation in high-speed trains [20] (figure 9*b*). Despite the different flow regime in suction-feeding fishes (laminar instead of turbulent flows in vehicles), the fluid-dynamic forces exerted on the ‘body’ of our models (i.e. the pectoral region, trunk and tail of the fish) showed differences of the same order of magnitude (maximally about 10%). Consequently, considering the fact that sharp edges are uncommon in animal bodies, our result that the morphology of the posterior pharynx may not be evolutionarily constrained by hydrodynamics in fishes may be analogous to road vehicle stylists' freedom to vary front-end shapes.

Given that the shape of the posterior pharynx appears to have a negligible effect on feeding hydrodynamics, why is there still a wedge-like shape in this region? The three-dimensional scan-reconstructions of our model species *Lepomis gibbosus* indeed showed a wedge-like protrusion formed by the cleithrum bone and the sternohyoideus muscle (figure 8). However, at the height of the oesophagus entrance and more dorsally towards the roof of the buccopharyngeal cavity, no such shape could be discerned. The main role of the sternohyoid muscle during powerful suction feeding is to pull the hyoid posteriorly by transmitting power from the considerably more forceful hypaxial muscles that retract the pectoral girdle. In some species, the sternohyoid muscle shortens during this phase [21], while in others it lengthens [22]. In either case, a key element in the expansion mechanism of the head is that sternohyoid force is applied on a midsagittal point on the hyoid arch, without interfering with the freedom of the left and right hyoid bars (i.e. the ceratohyals) to spread apart while pushing the suspensoria outwards [23]. This necessitates a narrow insertion site near the symphysis of the hyoid bars, which is provided by the urohyal bone and its ligamentous connection to the ceratohyals. Given the broader origin site at the cleithrum bone of the pectoral girdle, anterior tapering of the sternohyoid muscle seems inevitable. We, therefore, hypothesize that the wedge-like shape of the posterior–ventral border of the pharynx is mainly the result of mechanical demands to the force-transmission system from the pectoral girdle to the hyoid.

**Figure 10.**
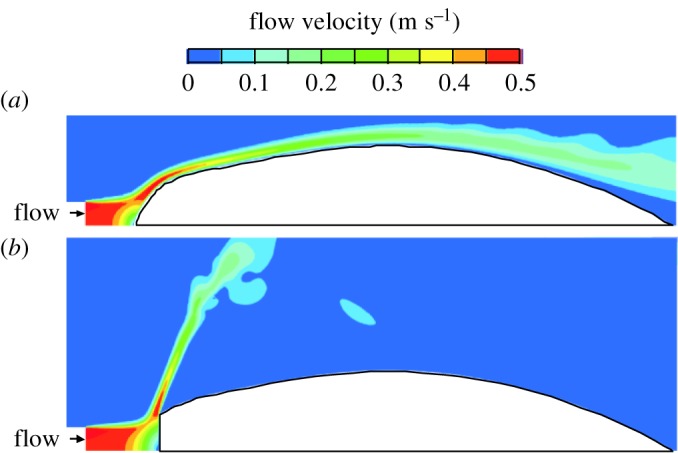
Models without buccal wall and gill cover subjected to a narrow stream impacting the front. The ‘body’ parts of rotationally symmetric models a1 (*a*) and a2 (*b*) (figure 3) show large differences in flow patterns: while in (*a*) the flow remains attached to the surface, in (*b*) the flow is deflected off the truncated anterior part. Note that the unfavourable situation of (*b*) is never observed in our fish models due to either the gill covers that influence the flow direction and/or the broader inflow zone.

We deliberately standardized the position and orientation of the gill cover in our models to exclusively quantify the influence of pharyngeal shape. Interestingly, while exploring different modelling approaches, the only simulation with a clearly different flow pattern was one in which the buccal cavity and gill cover surfaces were removed, and flow was forced to enter from a narrow zone at the mouth. In that case, a drastic deflection of the outflow was observed in the truncated model (figure 10*b*), whereas the bullet-shaped model showed flows that were nicely attached to the body (figure 10*a*). This example illustrates that the presence and orientation of the gill cover play a fundamental role in guiding the fluid out of the gill slits. In turn, this inevitably also influences the flow directions inside of the buccopharyngeal cavity. The role of the gill cover in suction-feeding hydrodynamics is worth exploring in further detail to better understand its functional morphology [24].

Several studies have previously demonstrated how the shape of different aspects of the suction-feeding apparatus is tuned to hydrodynamics. Examples are the shape of the mouth [25,26], the jaws [27,28], the snout [29,30] and the hyoid [31]. By contrast, the remarkable hydrodynamic insensitivity to shape modifications in the posterior region of the pharynx implies that hydrodynamic constraints on the morphology of this region are probably absent. The current study, therefore, showed that not all shapes subjected to fluid flows are the result of evolutionary optimization to improve fluid-dynamic performance.

## Supplementary Material

Table S1

## Supplementary Material

Table S2

## Data accessibility

Data relating to this work are available at the Dryad Digital Repository: http://dx.doi.org/10.5061/dryad.94jn4t9 [32].
