# Supplementary material for: Hydrodynamic performance of suction feeding is virtually unaffected by variation in the shape of the posterior region of the pharynx in fish
Source: R Soc Open Sci. 2018 Sep 19;5(9):181249. doi: 10.1098/rsos.181249 (PMC6170587; doi:10.1098/rsos.181249)
Supplement: Table S1 [file rsos181249supp1.docx]

**Table S1**

Values of pressure, velocity, wall shear and forces for the four static, rotationally symmetric, unsteady flow models three different time steps of the simulation (t_1_ = 8ms, t_2_ = 20 ms, t_3_ = 200 ms) calculated on the sagittal plane of the model, the body surface, gill cover surface and head surface (See Figure 2 for the description of the different shapes of the models and the location of the surfaces).
