# Supplementary material for: Hydrodynamic performance of suction feeding is virtually unaffected by variation in the shape of the posterior region of the pharynx in fish
Source: R Soc Open Sci. 2018 Sep 19;5(9):181249. doi: 10.1098/rsos.181249 (PMC6170587; doi:10.1098/rsos.181249)
Supplement: Table S2 [file rsos181249supp2.docx]

**Table S2**

| **Midsagittal plane** |  | **model c1** | **model c2** |  | **Difference** |
| --- | --- | --- | --- | --- | --- |
| Maximum Value of Pressure | [Pa] | 496.524 | 494.655 |  | -0.38% |
| Maximum Value of Velocity | [m/s] | 1.279 | 1.318 |  | 3.05% |
|  |  |  |  |  |  |
| **Frontal plane** |  | **model c1** | **model c2** |  | **Difference** |
| Maximum Value of Pressure | [Pa] | 401.594 | 483.836 |  | 20.48% |
| Maximum Value of Velocity | [m/s] | 1.208 | 1.224 |  | 1.33% |
|  |  |  |  |  |  |
| **Fish surface** |  | **model c1** | **model c2** |  | **Difference** |
| Maximum Value of Pressure | [Pa] | 500.814 | 501.499 |  | 0.14% |
| Maximum Value of Wall Shear | [Pa] | 70.835 | 73.850 |  | 4.26% |
| Axial Force | [N] | -0.00996 | -0.01002 |  | -0.60% |

Comparison of the maximum values of pressure and velocity calculated on the midsagittal and a frontal plane of the two static, 3D-scan based, steady flow models as well as maximum values of pressure and of wall shear, and value of axial force on the fish surface.
